# The Effects of Urbanization on Chronic Kidney Disease and Renal Function Decline: Findings from a Nation-Wide Longitudinal Study

**DOI:** 10.3390/toxics13110907

**Published:** 2025-10-23

**Authors:** Wei Liang, Dong Hou, Xiaoyu Li, Jiayi Qiu, Mei Wang, Xiuli Zhao, Shouxin Peng, Guangyu Lu

**Affiliations:** 1 School of Basic Medical Sciences & School of Public Health, Faculty of Medicine, Yangzhou University, Yangzhou 225009, China; 2 Zhenjiang Center for Disease Control and Prevention, 9# South Huangshan Road, Zhenjiang 212002, China; 3 Central China Subcenter of National Center for Cardiovascular Diseases, Zhengzhou 450046, China; 4 Jiangsu Key Laboratory of Zoonosis, Yangzhou University, Yangzhou 225009, China

**Keywords:** urbanization, chronic kidney disease, artificial nighttime light, average nighttime light index, nation-wide longitudinal study

## Abstract

Chronic kidney disease (CKD) has become a global public health concern, especially in developing countries. Although previous studies have suggested a link between urbanization and CKD, the existing evidence remains limited and inconsistent. We selected a sub-cohort of 5298 participants from the China Health and Retirement Longitudinal Study (CHARLS). All participants had normal renal function at baseline (2011) and were followed up in 2015, with renal biomarkers measured twice. Urbanization was assessed at the city level using the average nighttime light index (ANLI), derived from calibrated long-term satellite remote sensing data. Logistic regression models were used to examine the association between urbanization and CKD. Our results showed that a 0.1-unit increase in ANLI was associated with a 7.3% increase in the relative risk of CKD in the fully adjusted model (OR: 1.073, 95% CI: 1.045, 1.101). Subgroup analyses indicated that females (OR: 1.106, 95% CI: 1.068, 1.145) and urban residents (OR: 1.101, 95% CI: 1.060, 1.143) were at higher risk. We also found the synergistic amplification effects of heavier air pollution (PM_2.5_ and NO_2_) and elevated temperatures on this association. Our findings provide robust evidence of a positive association between urbanization and CKD among middle-aged and elderly adults in China. More scientific and specific health investment is needed with urbanization process simultaneously.

## 1. Introduction

Chronic kidney disease (CKD) is a global public health challenge, characterized by persistent urinary abnormalities, structural kidney damage, or impaired excretory function [1,2]. Over the past decade, global CKD-related deaths have increased by 33.7%, with projections indicating it will become the fifth leading cause of mortality by 2040 [3,4]. The disease burden is particularly heavy in China, with a prevalence of 10.8%, corresponding to approximately 119.5 million affected individuals [5]. The onset and progression of CKD are predominantly attributable to underlying conditions such as hypertension and diabetes, which contribute to renal damage through mechanisms like vascular injury and glomerular hyperfiltration. However, effective therapies for halting or reversing the progression of CKD, particularly in its advanced stages, remains limited. As CDK advances, patients often require dialysis or kidney transplantation, which impose significant financial burdens on individuals and healthcare systems [6]. Thus, comprehensively identifying modifiable CKD risk factors is urgent for precise prevention.

Urbanization has been increasingly recognized as a pivotal environmental determinant of health and a potential driver of CKD [7,8]. Rapid urbanization is linked to the rise in non-communicable diseases (NCDs) like hypertension and diabetes, which are established risk factors for CKD [9,10,11]. Concurrently, urban pollutants and environmental stressors are known to exacerbate the decline of renal function directly [12,13]. However, the direct evidence linking urbanization to CKD is both limited and inconsistent. A cross-sectional study from the UK reported that environmental urbanization was positively associated with CKD [14], whereas another study involving 1657 CKD patients reported that lower urbanization levels correlated with more severe CKD [15]. We hypothesize that one of the plausible explanations for the discrepancy may due to differences regarding the definition of urbanization. The above two studies evaluated the level of urbanization from different perspectives, anthropogenic pollution status [14] and socioeconomic status [15], which failed to comprehensively assess the impact of urbanization.

To address this limitation, satellite-derived artificial nighttime light data has emerged as a robust proxy for quantifying urbanization. The average nighttime light index (ANLI) quantifies the intensity of human activity by integrating data on economic output, population density, energy consumption, and infrastructure development [16,17,18,19,20], thereby offering a more comprehensive measure. Notably, a nationwide cross-sectional study in China showed that each 10-unit increase in the five-year ANLI was associated with a 5% higher prevalence of CKD [21], providing preliminary evidence for its relevance. Beyond its utility as a measurement tool, nighttime light exposure is itself a recognized circadian disruptor associated with obesity [22], cardiovascular disease [23], and cancer [24], and represents a plausible biological pathway for CKD. Despite this, longitudinal data exploring the link between ANLI-quantified urbanization and CKD incidence, as well as renal function decline, are lacking, leaving a critical gap in understanding of urbanization’s role in CKD progression.

China is experiencing rapid urbanization, which intensifies environmental health burdens and complicates CKD prevention [25] Therefore, we conducted a nationwide longitudinal study to comprehensively investigate the effect of urbanization, quantified by ANLI, on CKD and renal function decline. We hypothesize that higher levels of urbanization are associated with an increased risk of CKD and accelerated renal function decline, and that this relationship is significantly mediated by the increased prevalence of cardiometabolic diseases such as hypertension and diabetes.

## 2. Materials and Methods

### 2.1. Study Design and Population

The current longitudinal study is based on the China Health and Retirement Longitudinal Study (CHARLS), which investigated a multi-center dynamic cohort of Chinese residents aged over 45 years. Participants were selected through a multistage probability sampling method covering 150 counties across 28 provinces in China. All respondents were followed up every two years, with detailed information collected on their social, economic, and health conditions [26]. To date, four rounds of follow-up surveys have been conducted, including the baseline survey in 2011 (Wave 1). We analyzed the data from Wave 1 and Wave 3, which include blood sample collection and biomechanical analysis of blood biomarkers, specifically serum creatinine and fasting glucose, measured using the rate-blanked and compensated Jaffe method and an enzymatic colorimetric test, respectively. Wave 1 included 11,847 participants with biomarker data, in which 7648 subjects had repeated measurements in Wave 3. We further selected the participants with complete demographic, lifestyle, health status, and physical measurement data in Wave 1 (*n* = 6327). We then excluded individuals with missing serum creatinine measurements in either wave, as well as those with diagnosed kidney disease, cancer, or other serious conditions at baseline. Finally, a total of 5298 participants were included in the final longitudinal cohort, with detailed information on exclusions (including numbers per category) provided in Supplementary Figure S1.

### 2.2. Urbanization Level Assessment

We used the nighttime light index (NLI) as an indicator to quantify the levels of urbanization. Traditional NLI datasets of DMSP-OLS (annual mean values from 1992 to 2013) and NPP-VIIRS (monthly mean values since April 2012) differ in sensor design and spatial resolutions. Chen et al. proposed a cross-sensor calibration method using a vegetation index and an auto-encoder model with a good consistency at the city level (*R*^2^ = 0.95) and producing extended NPP-VIIRS-like nighttime light data for 2000–2018 [27]. Daily temperature data were obtained from 699 meteorological monitoring station in Chinese Surface Daily Climate Dataset version 3.0, provided by the China Meteorological Data Service Center (https://data.cma.cn/en/, accessed on 3 June 2024). We manually rechecked and rectified all the suspicious or incorrect data to enhance the homogeneity and reliability. Daily averaged temperature was interpolated for each city where the participants are resident, using the inverse distance weighted (IDW) [28]. Ambient fine particulate matter (PM_2.5_) and nitrogen dioxide (NO_2_) concentrations were calculated using random forest models that incorporated meteorological data, aerosol optical depth, land use information, and ground monitoring concentrations [29,30].

### 2.3. Health Outcomes

Renal function was quantified using the estimated glomerular filtration rate (eGFR), which was calculated by four key parameters (age, gender, race, and serum creatinine) following the Chronic Kidney Disease Epidemiology Collaboration equation [31] Participants with an eGFR < 60 mL/min/1.73 m^2^ were defined as an incident of CKD. We also calculated the decline of eGFR using the formula (eGFRWave1-eGFRWave3)/eGFRWave1 × 100%. An eGFR decline greater than 30% was defined as another outcome indicator closely associated with higher risks of end-stage renal disease [32].

### 2.4. Covariates

All covariates were collected from the baseline survey in Wave 1. Age (years) and gender (male or female) were obtained from questionnaires. Body mass index (BMI) was calculated as height divided by weight squared (kg/m^2^). Marital status was categorized as “Married” and “Separated/Divorced/Widowed”. Education levels were classified with “Illiteracy” and “Elementary school or above”. Drinkers were defined as those who had drunk in the past 12 months, while smokers were defined according to whether they had smoked more than 100 cigarettes in their lifetime. Current residence was divided into rural and urban according to the community ID, consistent with China’s National Administrative Division Standards (GB/T 2260–2007) [33]. Hypertension was defined as meeting any of the following criteria: (i) systolic blood pressure > 140 mmHg, (ii) diastolic blood pressure > 90 mmHg, (iii) having been diagnosed with hypertension by a clinical doctor, and (iv) the use of anti-hypertensive medication. We defined diabetes as fasting glucose greater than 200 mg/dL, being clinically diagnosed with diabetes, and/or the intake of antidiabetic medication. NCDs of cardiovascular disease and stroke were defined according to self-reported status in questionnaire, while glucose was obtained from blood biomarker examination.

### 2.5. Statistical Analyses

Baseline characteristics involving demographic factors and health status of the overall participants were described as mean (standard deviation) or frequency (percentage). We performed the generalized linear models to explore the association between urbanization levels and the risk of CKD and renal function decline. The 5-year average ANLI was calculated over the entire study period. Four regression models were developed: the Crude model was unadjusted; Model I was adjusted age, gender, BMI, living residence, marriage status, and educational status; Model II was further adjusted for 4 kinds of NCDs (hypertension, diabetes, stroke, and cardiovascular disease) and the baseline eGFR levels; and Model III (the main model) was additionally adjusted for annual averaged temperature and concentrations of PM_2.5_ and NO_2_. We assessed multicollinearity among the independent variables using variance inflation factors (VIFs), with a VIF value less than 10 considered indicative of no significant multicollinearity. Subgroup analyses were performed by age (45–65 or ≥65 years), gender, BMI (<24 or ≥24 kg/m^2^), lifestyles (drinking and smoking), NCDs (hypertension or diabetes), residence, and the median of environment factor (PM_2.5_ and NO_2_ concentration and temperature). Differences between subgroups were tested using the *Z*-test. Furthermore, the interaction relationship among CKD, environmental factors, and ANLI was visualized using 3D surface fitting. All statistical analyses were conducted using R 4.2.0 (R Foundation for Statistical Computing, Vienna, Austria). The statistical significance was defined as a two-sided *p*-value less than 0.05.

## 3. Results

### 3.1. Descriptive Analyses

**Figure 1:**
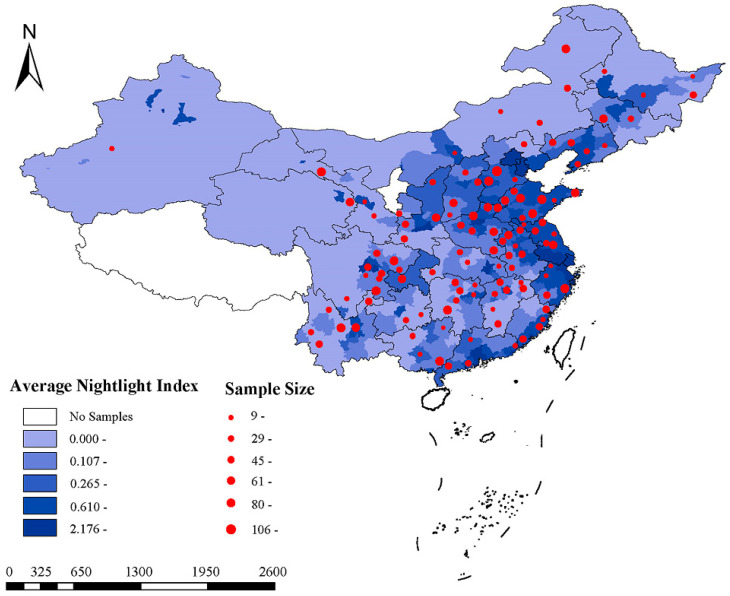
Spatial distribution of 5-year average nightlight index from 2011 to 2015 and residential address in the current study.

A total of 5298 participants (2395 males and 2903 females) were included, with a mean age of 58.6 years and a mean BMI of 23.6 kg/m^2^ at baseline in 2011. Among these participants, 32.6% reported alcohol consumption habits and 37.9% reported tobacco use habits, respectively. The proportion of married participants was 88.8%, and 68.9% of the participants resided in a rural area (Table 1). The spatial distribution of the 5-year ANLI from 2011 to 2015 and the residential addresses of the participants in this study are shown in Figure 1. The 5-year ANLI across the 113 Chinese cities included in the study range from 0.017 to 2.176, with a mean of 0.438 U. The mean annual ambient temperature was 15.4 °C. Additionally, the average concentrations of PM_2.5_ and NO_2_ during the study periods were 53.3 μg/m^3^ and 28.4 μg/m^3^, respectively. After a nearly 5-year follow-up, 242 participants were diagnosed with CKD, and 230 experienced an eGFR decline greater than 30%. We found the participants who were older, had a lower baseline eGFR, or suffered from hypertension or diabetes, were more likely to develop CKD. Furthermore, higher ambient temperature might also be a risk factor for both CKD and renal function decline (Table S1).

### 3.2. Regression Analyses

In the fully adjusted model, a 0.1 U increase in ANLI was associated with a 7.3% higher risk of incident CKD (OR: 1.073, 95% CI: 1.045, 1.101) and a 7.0% higher risk of an eGFR decline greater than 30% (OR: 1.070, 95% CI: 1.042, 1.097), respectively (Table 2). To verify model robustness, VIF assessment for the fully adjusted model showed all values <2, indicating no significant multicollinearity and stable regression results. Furthermore, ANLI was categorized into quartiles based on its distribution in the study population to explore potential dose–response relationships, with cutoffs derived from the 25th, 50th, and 75th percentiles of the ANLI distribution. Compared with participants in the lowest ANLI quartile, those in the third and fourth quantiles had a significantly higher risk of developing CKD, with ORs of 2.622 (95% CI: 1.610, 4.324) and 2.521 (95% CI: 1.573, 4.092), respectively. For an eGFR decline greater than 30%, the highest risk was observed in participants in the fourth ANLI quartile (OR: 2.528, 95% CI: 1.596, 4.046).

### 3.3. Stratification Analyses

**Figure 2:**
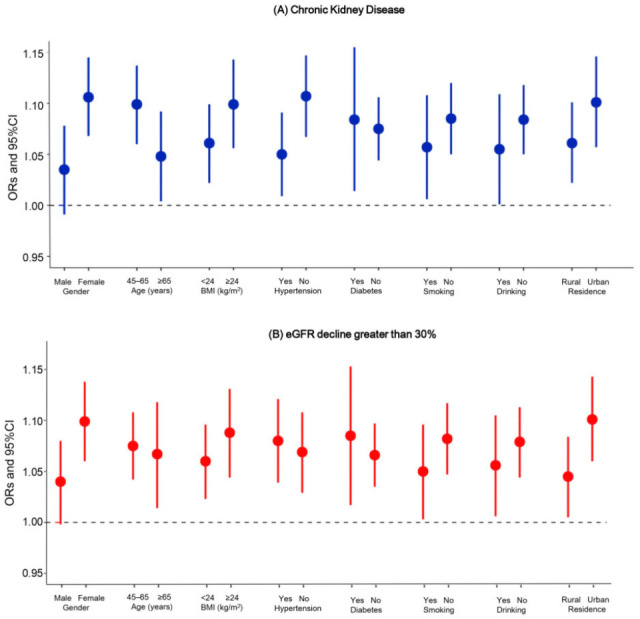
The stratified analyses of the association between ANLI and CKD or an eGFR decline greater than 30%. Abbreviations: eGFR, estimated glomerular filtration rate; ORs, odds ratios; CI, confidence interval.

The effects of ANLI on CKD and eGFR decline were modified by gender, hypertension, residential location, temperature, and air pollutants (Figure 2). In the multivariable-adjusted model, the OR for incident CKD was 1.106 (95% CI: 1.068, 1.145) in females, significantly higher than that in males (OR: 1.035, 95% CI: 0.991, 1.078; *p* for interaction = 0.01). A similar gender-dependent difference was also observed for the association between ANLI and eGFR decline >30%.

**Figure 3:**
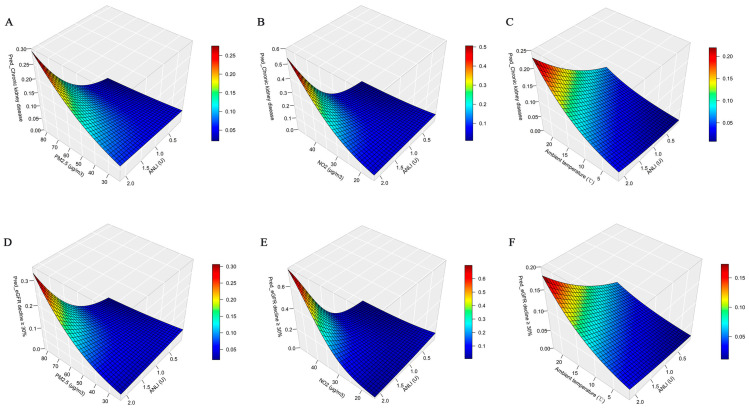
The 3D curved surface of the mutual effect of ANLI, ambient temperature, and air pollution (PM_2.5_ and NO_2_ concentration) on CKD and an eGFR decline greater than 30%. In Figure 3, (**A**–**C**) presents the relevant results of CKD; (**D**–**F**) presents the relevant results of eGFR decline greater than 30%. Pred_Chronic kidney disease and Pred_eGFR decline ≥ 30% are the predictive values of ANLI and environmental factors (temperature, PM_2.5_, and NO_2_) for CKD and eGFR decline greater than 30%, respectively. Abbreviations: ANLI, average nightlight index; PM_2.5_, fine particulate matter; NO_2_, nitrogen dioxide.

Compared with the participants residing in rural areas (OR: 1.045, 95% CI: 1.005, 1.084), those in urban areas had a higher risk of an eGFR decline greater than 30% (OR: 1.101, 95% CI: 1.060, 1.143). Additionally, the predicted risks of incident CKD and an eGFR decline >30% increased with increasing levels of ambient temperature, NO_2_, PM_2.5_, and ANLI (Figure 3). Stratified analyses further indicated that the effect of ANLI on both CKD and eGFR decline was more pronounced under higher ambient temperatures and air pollutant levels (Table S2).

### 3.4. Sensitivity Analyses

A series of sensitivity analyses were conducted to examine the robustness of our findings. Firstly, we further adjusted NDVI, which was recognized as an environmental indicator strongly linked to health, into the fully adjusted model (Model III). These results were also consistent with our primary observations (Table S3). In addition, we calculated the 3-year-averaged ANLI levels to examine the association between urbanization with CKD and renal function decline. The positive association of ANLI with CKD and eGFR decline greater than 30% remain significant (Table S4). Finally, we replaced the CKD with eGFR from Wave 3 into the multivariate linear regression models. Significant associations between ANLI and eGFR decline were observed across all five exposure windows (Figure S2), supporting the consistency of our main results.

## 4. Discussion

This nationwide longitudinal study demonstrates that higher levels of urbanization, as quantified by the ANLI, are associated with increased odds of CKD and accelerated renal function decline among middle-aged and elderly Chinese adults. Notably, the adverse effect of ANLI on renal function decline was more pronounced in females and those residing in urban areas. Our findings contribute to the existing literature by being the first, to our knowledge, to employ ANLI as a comprehensive, objective proxy for urbanization in a longitudinal assessment of kidney health.

Previous studies have provided the evidence urbanization and kidney disease from different perspectives [8,24]. Studies utilizing socioeconomic indicators [5], composite indices [8], and nighttime light data [24] have similarly reported positive associations. From the perspective of economic development, a cross-sectional study among 50,500 Chinese adults reported that higher social economic levels were positively associated with renal damage [5]. Another research from the CHNS also reported that the positive association between urbanization and CKD in women and men, using a 12-component composite index [8]. Besides, the China National Survey of CKD reported that a 10-unit increase in NLI was associated with a higher risk of CKD (OR: 1.05, 95% CI: 1.02, 1.07) [24]. Our findings are consistent with the three aforementioned studies. However, the literature is not entirely consistent, with some studies reporting higher CKD odds in urban areas [34], and others in rural regions [35]. We posit that these discrepant findings largely stem from the use of inconsistent and often oversimplified definitions of urbanization, such as administrative binary classifications (urban/rural), which lack comparability across different national contexts. The ANLI, in contrast, offers a standardized, continuous measure that captures the multifaceted nature of urban development, thereby helping to resolve these inconsistencies.

The mechanisms underpinning the association between urbanization and kidney disease are multifactorial and not yet fully elucidated or understood. Several plausible hypotheses have been proposed to explain this relationship. One hypothesis suggests that rapid and unplanned urbanization contributes to environmental pollution [36] and climate change [37], which are recognized as the risk factors of CKD [13]. Another hypothesis indicates that rapid urbanization would provoke series of adverse lifestyle changes [7,38] and increase the prevalence of numerous chronic diseases (hypertension, diabetes, and obesity) [39,40], which in turn contribute to kidney disease development. Our causal mediation analysis provides empirical support for one such pathway, indicating that hyperglycemia mediates the effect of urbanization on both incident CKD and renal function decline (Figure S3). Furthermore, artificial light at night, a hallmark of urbanization, may exert additional neuroendocrine effects that promote obesity and cardiovascular disease, potentially compounding the risk of renal impairment [21,22,41].

Beyond direct mechanisms, effect modification by environmental co-exposures appears to intensify the urban–renal link. Air pollution, particularly PM_2.5_ and NO_2_, is not only a recognized renal hazard but also frequently co-occurs with urban development [37,42,43,44]. Our results demonstrate a stronger association between urbanization and CKD in subgroups with higher exposure to air pollution, suggesting a synergistic amplification of risk. This is visually supported by our 3D surface analysis and aligns with prior reports of interaction between these exposures [24]. We hypothesize that pre-existing subclinical renal injury induced by air pollutants [45,46] may heighten susceptibility to other urban stressors. Elevated ambient temperature represents another significant modifier. It may impair renal function directly by stimulating vasopressin release and sympathetic activity, reducing renal perfusion [47], and indirectly by fostering unhealthy behaviors linked to urbanization, such as physical inactivity and poor dietary choices [48], which may collectively worsen kidney injury.

We observed the modification effect of gender and residential location on the association between urbanization and kidney disease. The association was stronger in females, and we propose two potential mechanisms for this gender difference. First, women may constitute a more vulnerable subgroup to urbanization-related air pollution, whereas the higher prevalence of smoking among men may confound or mask the true association in this group [49]. Second, urbanization-related reductions in estrogen levels can induce glomerulosclerosis and tubulointerstitial fibrosis [50], which further contribute to the higher risk for kidney disease among female [51]. In addition, the association for urbanization and renal function decline was generally stronger in urban residents. Although better healthcare access and health literacy in highly urbanized areas are theoretically protective against chronic diseases [1,52], these advantages may be offset by intensified exposure to environmental pollutants and lifestyle risk factors, such as physical inactivity, which likely exacerbate kidney injury [8,53].

Our study has a few strengths. Firstly, this was a nation-wide longitudinal study in 111 of China’s cities to explore the association between urbanization and CKD and renal function decline, which provide the novel epidemiological evidence on the health effect of urbanization. Secondly, to our knowledge, this is the first study to apply the cities’ ANLI obtained from remote sensing to quantity the level of urbanization for health risk assessment in China. And the calibrated long-term time series nighttime light data could be used for subsequent urbanization-related studies. Thirdly, we conducted series of stratified analyses, mediation effect analyses, and sensitivity analyses, which revealed the interactions between environmental factors (air pollution and climate change) and urbanization, and further corroborate the reliability of our findings. These findings have significant public health implications. They underscore the need to integrate kidney disease prevention into urban planning and environmental policy. Strategies aimed at mitigating air pollution, promoting physical activity, and ensuring healthy food environments in rapidly urbanizing areas are crucial.

There are also several limitations in the current study. Firstly, since the main research subjects in CHARLS were distributed in the central and eastern regions of China [27], there are fewer research subjects in the western region in this study. Additionally, we also excluded several cities with extreme values of ANLI in the statistical regression models, which might limit our findings extensionality. Secondly, participants from the current research were middle-aged and elderly adults over 45 years. Due to their established lifestyle habits, heightened health consciousness, and limited adoption of novel urban services, their behavioral patterns were less likely to be influenced by urbanization. This may have led us to underestimate the impact of urbanization on kidney disease prevalence. Thirdly, kidney function was assessed using eGFR rates based on serum creatinine, which subject to measurement variability and does not incorporate albuminuria data. This approach likely resulted in the underdetection of early-stage CKD and may have led to underestimated effect sizes. Finally, we applied the simplified criterion for CKD definition due to complexity of the clinical diagnostic guidelines for CKD published by the KDIGO CKD Work Group [54]. Our current criterion may lead to the misclassification of CKD. However, the criteria for defining CKD in previous studies were inconsistent. The criterion in a US cohort was determined as two eGFR < 60 mL/min/1.73 m^2^ measurements at least 90 days apart [43], while Li et al. defined CKD with albuminuria or eGFR < 60 mL/min/1.73 m^2^ [55]. And there have also several studies based on our current definition [56,57]. Future studies are needed to evaluate the differences in findings under different definition criteria.

## 5. Conclusions

This longitudinal study establishes a significant association between urbanization and both CKD incidence and renal function decline in middle-aged and elderly Chinese adults, with stronger effects observed in females, urban residents, and populations exposed to higher air pollution or temperatures. These findings highlight the need to incorporate health considerations into urban planning. Future research should focus on elucidating the underlying mechanisms and validating these associations through multigenerational cohort studies.

## Figures and Tables

**Table 1:** Descriptive Statistics of the Study Participants.

Characteristic		Mean ± SD or N (%)
NO.		5298
Age (years)		58.6 ± 8.5
Gender (n, %)		
	Male	2395 (45.2)
	Female	2903 (54.8)
BMI (kg/m^3^)		23.6 ± 3.8
Education level (n, %)		
	Illiteracy	4850 (91.5)
	Elementary school or above	448 (8.5)
Marital status (n, %)		
	Married	4705 (88.8)
	Separated/Divorced/Widowed	593 (11.2)
Smoking status (n, %)		
	Smoker	2008 (37.9)
	Non-smoker	3290 (62.1)
Drinking status (n, %)		
	Drinker	1727 (32.6)
	Non-drinker	3571 (67.4)
Current residence (n, %)		
	Rural	3649 (68.9)
	Urban	1649 (31.1)
Hypertension (n, %)		2080 (39.3)
Diabetes (n, %)		702 (13.3)
Cardiovascular disease (n, %)		585 (11.0)
Stroke (n, %)		97 (1.8)
Baseline eGFR (mL/min/1.73 m^2^)		96.2 ± 14.6
Temperature (°C)		15.4 ± 4.3
PM_2.5_ (μg/m^3^)		53.3 ± 13.6
NO_2_ (μg/m^3^)		28.4 ± 8.4

Abbreviations: BMI, body mass index; PM_2.5_, fine particulate matter; NO_2_, nitrogen dioxide.

**Table 2:** Association between ANLI and CKD or eGFR decline greater than 30%.

		CKD		eGFR Decline Greater than 30%	
		OR (95% CI)	*p*	OR (95% CI)	*p*
Continuous variables					
	Crude model	1.080 (1.055, 1.105)	<0.001	1.067 (1.041, 1.092)	<0.001
	Model I	1.077 (1.050, 1.103)	<0.001	1.067 (1.040, 1.093)	<0.001
	Model II	1.080 (1.053, 1.107)	<0.001	1.068 (1.041, 1.095)	<0.001
	Model III	1.073 (1.045, 1.101)	<0.001	1.07 (1.042, 1.097)	<0.001
Categorical variables					
	Q1 (0.017~0.107)	ref.		ref.	
	Q2 (0.107~0.265)	1.082 (0.663, 1.773)	0.754	1.421 (0.893, 2.276)	0.140
	Q3 (0.265~0.610)	2.622 (1.610, 4.324)	<0.001	2.402 (1.480, 3.935)	<0.001
	Q4 (0.610~2.176)	2.521 (1.573, 4.092)	<0.001	2.528 (1.596, 4.046)	<0.001

Abbreviations: ANLI, average nightlight index; CKD, chronic kidney disease; eGFR, estimated glomerular filtration rate; OR, odds ratio; CI, confidence interval. Crude model unadjusted; Model I adjusted for age, gender, BMI, residence, marital status, and educational status; Model II further adjusted for hypertension, diabetes, stroke, cardiovascular disease, and baseline eGFR levels; Model III further adjusted for annual averaged temperature and concentrations of PM_2.5_ and NO_2_.

## Data Availability

The data presented in this study are openly available from the China Health and Retirement Longitudinal Study (CHARLS) at https://charls.pku.edu.cn/ (accessed on 16 December 2023).

## Supplementary Materials

The following supporting information can be downloaded at: https://www.mdpi.com/article/10.3390/toxics13110907/s1, Figure S1. Flow chart of the selection process of the participants. Table S1. Difference in the baseline characteristics of participants with (or without) developed chronic kidney disease and eGFR decline greater than 30%. Table S2. The stratified analyses of the association between ANLI and chronic kidney disease or eGFR decline greater than 30%. Table S3. Association between ANLI and chronic kidney disease or eGFR decline greater than 30% with a further adjusted NDVI. Table S4. Association between 3-year ANLI and chronic kidney disease or eGFR decline greater than 30%. Figure S2. Association between ANLI and eGFR in different exposure windows. Figure S3. Mediation of association between ANLI and chronic kidney disease or eGFR decline greater than 30% by blood glucose.

## Abbreviations

The following abbreviations are used in this manuscript:

PM_2.5_	fine particulate matter
NO_2_	nitrogen dioxide
NCDs	non-communicable disease
CKD	chronic kidney disease
CHARLS	China Health and Retirement Longitudinal Study
ANLI	average nighttime light index
NLI	nighttime light index
IDW	inverse distance weighted
BMI	body mass index
eGFR	estimated glomerular filtration rate
OR	odds ratio
CI	confidence interval
