# Supplementary material for: The Effects of Urbanization on Chronic Kidney Disease and Renal Function Decline: Findings from a Nation-Wide Longitudinal Study
Source: Toxics. 2025 Oct 23;13(11):907. doi: 10.3390/toxics13110907 (PMC12656146; doi:10.3390/toxics13110907)
Supplement: Supplementary file 1 [file toxics-13-00907-s001.zip › toxics-3913893-SI.pdf]

Supplement Information

The Effects of Urbanization on Chronic Kidney Disease and Renal Function Decline: Findings from a Nation-Wide Longitudinal Study

Figure S1. Flow chart of the selection process of the participants.

Table S1. Difference in the baseline characteristics of participants with (or without) developed chronic kidney disease and eGFR decline greater than 30%.

Table S2. The stratified analyses of the association between ANLI and chronic kidney disease or eGFR decline greater than 30%.

Table S3. Association between ANLI and chronic kidney disease or eGFR decline greater than 30% with further adjusted the NDVI.

Table S4. Association between 3-year ANLI and chronic kidney disease or eGFR decline greater than 30%.

Figure S2. Association between ANLI and eGFR in different exposure windows.

Figure S3. Mediation of association between ANLI and chronic kidney disease or eGFR decline greater than 30% by blood glucose.

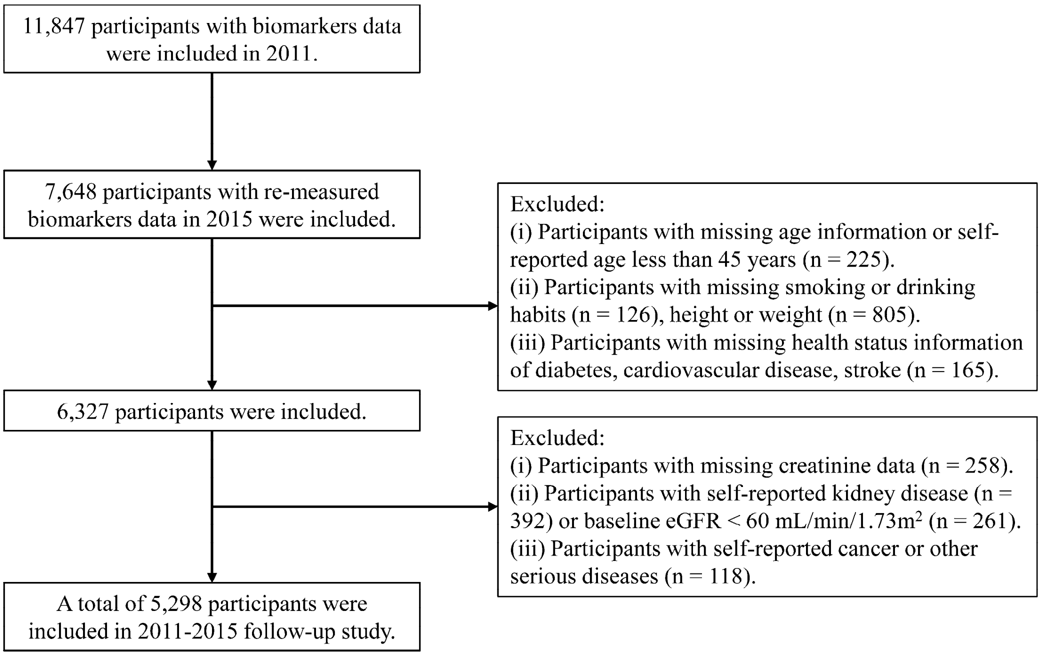

Figure S1. Flow chart of the selection process of the participants.

Table S1. Difference in the baseline characteristics of participants with (or without) developed chronic kidney disease and eGFR decline greater than 30%.

Characteristic	Development of CKD			Incidence of eGFR decline greater than 30%		
	Yes (n=242)	No (n=5056)	*P*	Yes (n=230)	No (n=5068)	*P*
Age (years)	64.7 ± 8.6	58.3 ± 8.4	<0.001	60.7 ± 8.7	58.5 ± 8.5	<0.001
Gender (n, %)			0.644			0.042
Male	113 (46.7%)	2282 (45.1%)		119 (51.7%)	2276 (44.9%)	
Female	129 (53.3%)	2774 (54.9%)		111 (48.3%)	2792 (55.1%)	
BMI (kg/m^3)	23.8 ± 3.6	23.6 ± 3.8	0.367	23.8 ± 3.7	23.6 ± 3.8	0.478
Education level (n, %)			<0.001			0.021
Illiteracy	236 (97.5%)	4614 (91.3%)		220 (95.7%)	4630 (91.4%)	
Elementary school or above	6 (2.5%)	442 (8.7%)		10 (4.3%)	438 (8.6%)	
Marital status (n, %)			<0.001			0.054
Married	47 (19.4%)	546 (10.8%)		195 (84.8%)	4510 (89.0%)	
Separated/Divorced/Widowed	195 (80.6%)	4510 (89.2%)		35 (15.2%)	558 (11.0%)	
Smoking status (n, %)			0.735			0.144
Smoker	89 (36.8%)	1919 (38.0%)		98 (42.6%)	1910 (37.7%)	
Non-smoker	153 (63.2%)	3137 (62.0%)		132 (57.4%)	3158 (62.3%)	
Drinking status (n, %)			0.44			0.315
Drinker	73 (30.2%)	1654 (32.7%)		82 (35.7%)	1645 (32.5%)	
Non-drinker	169 (69.8%)	3402 (67.3%)		148 (64.3%)	3423 (67.5%)	
Current residence (n, %)			0.019			0.513
Rural	150 (62.0%)	3499 (69.2%)		154 (67.0%)	1573 (31.0%)	
Urban	92 (38.0%)	1557 (30.8%)		76 (33.0%)	3495 (69.0%)	
Hypertension (n, %)	129 (53.3%)	1951 (38.6%)	<0.001	118 (51.3%)	1962 (38.7%)	<0.001

Diabetes (n, %)	43 (17.8%)	659 (13.0%)	0.041	41 (17.8%)	661 (13.0%)	0.046
Cardiovascular disease (n, %)	35 (14.5%)	550 (10.9%)	0.092	26 (11.3%)	559 (11.0%)	0.914
Stroke (n, %)	9 (3.7%)	88 (1.7%)	0.042	6 (2.6%)	91 (1.8%)	0.316
Baseline eGFR (mL/min/1.73m^2)	82.1 ± 15.4	96.9 ± 14.2	<0.001	99.1 ± 16.3	96.1 ± 14.5	0.007
Temperature (˚C)	16.9 ± 3.5	15.3 ± 4.3	<0.001	16.6 ± 3.8	15.4 ± 4.3	<0.001
$PM_{2.5}$ (μg/m^3)	53.9 ± 12.0	53.3 ± 13.7	0.443	53.8 ± 11.2	53.3 ± 13.7	0.549
NO_2 (μg/m^3)	28.1 ± 7.9	28.4 ± 8.4	0.647	27.9 ± 7.5	28.4 ± 8.4	0.318

Abbreviations: CKD, chronic kidney disease; eGFR, estimated glomerular filtration rate; $PM_{2.5}$, fine particulate matter; NO_2, nitrogen dioxide.

Table S2. The stratified analyses of the association between ANLI and chronic kidney disease or eGFR decline greater than 30%.

	N	Chronic Kidney Disease			eGFR decline greater than 30%		
		OR	95%CI	*P*-value*	OR	95%CI	*P*-value*
Temperature				0.042			0.044
Low	2619	0.960	(0.848, 1.069)		0.845	(0.845, 1.063)	
High	2679	1.083	(1.054, 1.113)		1.049	(1.049, 1.107)	
$PM_{2.5}$ concentration				0.003			<0.001
Low	2634	1.004	(0.944, 1.062)		0.907	(0.907, 1.038)	
High	2664	1.104	(1.069, 1.139)		1.081	(1.081, 1.153)	
NO_2 concentration				<0.001			<0.001
Low	2651	1.003	(0.958, 1.046)		0.910	(0.910, 1.008)	
High	2647	1.148	(1.104, 1.194)		1.125	(1.125, 1.215)	

Notes: * *P*-values for stratified analysis by Z-test. Abbreviations: ANLI, average nightlight index; eGFR, estimated glomerular filtration rate; $PM_{2.5}$, fine particulate matter; NO_2, nitrogen dioxide. The multivariable adjusted model adjusted for age, gender, body mass index, living residence, marriage status and education status, hypertension, diabetes, stroke, cardiovascular disease, the baseline eGFR levels, and the annual averaged-temperature and concentrations of $PM_{2.5}$ and NO_2.

Table S3. Association between ANLI and chronic kidney disease or eGFR decline greater than 30% with further adjusted the NDVI.

	CKD			eGFR decline greater than 30%		
	OR	95%CI	*P*	OR	95%CI	*P*
Continuous variables						
Model IV	1.075	(1.046, 1.103)	<0.001	1.071	(1.043, 1.098)	<0.001
Categorical variables						
Q1 (0.017-0.107)	ref.			ref.		
Q2 (0.107-0.265)	1.095	(0.668, 1.803)	0.720	1.462	(0.913, 2.359)	0.116
Q3 (0.265-0.610)	2.636	(1.617, 4.352)	<0.001	2.444	(1.502, 4.016)	<0.001
Q4 (0.610-2.176)	2.556	(1.585, 4.174)	<0.001	2.611	(1.634, 4.215)	<0.001

Abbreviations: ANLI, average nightlight index; NDVI, normalized differential vegetation index; CKD, chronic kidney disease; eGFR, estimated glomerular filtration rate. Model IV adjusted for age, gender, BMI, habits of drinking and smoking, living residence, marriage status, education, stroke, cardiovascular disease, hypertension, diabetes, baseline eGFR, NO_2 concentrations, $PM_{2.5}$ concentrations, mean temperature and the NDVI.

Table S4. Association between 3-year ANLI and chronic kidney disease or eGFR decline greater than 30%.

	CKD			eGFR decline > 30%		
	OR	95%CI	*P*	OR	95%CI	*P*
Continuous variables						
Crude model	1.064	(1.045, 1.084)	<0.001	1.054	(1.033, 1.074)	<0.001
Model I	1.062	(1.041, 1.082)	<0.001	1.054	(1.032, 1.074)	<0.001
Model II	1.063	(1.042, 1.085)	<0.001	1.056	(1.034, 1.077)	<0.001
Model III	1.056	(1.034, 1.079)	<0.001	1.053	(1.031, 1.075)	<0.001
Categorical variables						
Q1 (0.011-0.146)	ref.			ref.		
Q2 (0.146-0.332)	0.861	(0.528, 1.409)	0.549	0.878	(0.541, 1.421)	0.596
Q3 (0.332-0.715)	2.294	(1.396, 3.815)	0.001	2.111	(1.307, 3.442)	0.002
Q4 (0.715-2.983)	2.211	(1.365, 3.622)	0.001	2.070	(1.302, 3.314)	0.002

Abbreviations: ANLI, average nightlight index; NDVI, normalized differential vegetation index; CKD, chronic kidney disease; eGFR, estimated glomerular filtration rate. Crude model unadjusted; Model I adjusted for age, gender, body mass index, living residence, marriage status and education status; Model II further adjusted hypertension, diabetes, stroke, cardiovascular disease and the baseline eGFR levels; Model III further adjusted annual averaged-temperature and concentrations of $PM_{2.5}$ and NO_2.

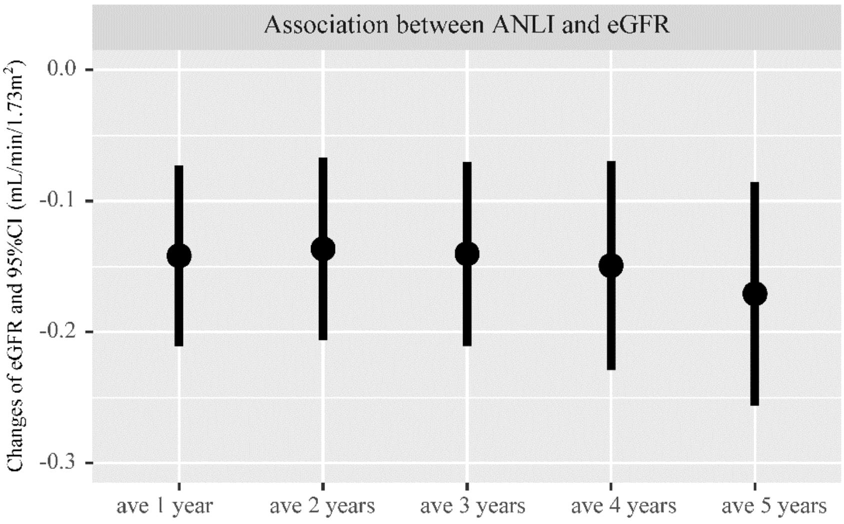

Figure S2. Association between ANLI and eGFR in different exposure windows. Abbreviations: ANLI, average nightlight index; eGFR, estimated glomerular filtration rate. The multivariable linear regression model adjusted for age, gender, body mass index, living residence, marriage status and education status, hypertension, diabetes, stroke, cardiovascular disease, the baseline eGFR levels, and the annual averaged-temperature and concentrations of $PM_{2.5}$ and NO_2.

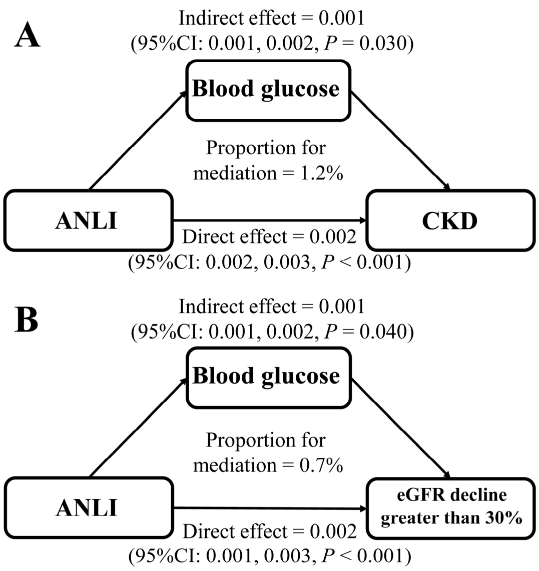

Figure S3. Mediation of association between ANLI and chronic kidney disease or eGFR decline greater than 30% by blood glucose. The causal mediation analysis following the Baron-Kenny's step to examine whether cardiometabolic function could be mechanisms through which urbanization affected renal function. Briefly, we included blood glucose and blood pressure as the potential bio-mediators in the model, respectively. And the results were generated using the R packages of "mediation" with bootstrapping of 1000 simulations.
